# Large-scale protein function prediction using heterogeneous ensembles

**DOI:** 10.12688/f1000research.16415.1

**Published:** 2018-09-28

**Authors:** Linhua Wang, Jeffrey Law, Shiv D. Kale, T. M. Murali, Gaurav Pandey

**Affiliations:** 1 Department of Genetics and Genomic Sciences and Icahn Institute for Genomics and Multiscale Biology, Icahn School of Medicine at Mount Sinai, New York, NY, 10029, USA; 2 Genetics, Bioinformatics, and Computational Biology Ph.D. Program, Virginia Polytechnic Institute and State University, Blacksburg, VA, 24061, USA; 3 Biocomplexity Institute, Virginia Polytechnic Institute and State University, Blacksburg, VA, 24061, USA; 4 Department of Computer Science, Virginia Polytechnic Institute and State University, Blacksburg, VA, 24061, USA

**Keywords:** protein function prediction，heterogeneous ensembles，machine learning， high-performance computing， performance evaluation

## Abstract

Heterogeneous ensembles are an effective approach in scenarios where the ideal data type and/or individual predictor are unclear for a given problem. These ensembles have shown promise for protein function prediction (PFP), but their ability to improve PFP at a large scale is unclear. The overall goal of this study is to critically assess this ability of a variety of heterogeneous ensemble methods across a multitude of functional terms, proteins and organisms. Our results show that these methods, especially Stacking using Logistic Regression, indeed produce more accurate predictions for a variety of Gene Ontology terms differing in size and specificity. To enable the application of these methods to other related problems, we have publicly shared the HPC-enabled code underlying this work as LargeGOPred ( https://github.com/GauravPandeyLab/LargeGOPred).

## Introduction

Given the large and rapidly growing gap between sequenced genomes and experimentally determined functional annotations of the constituent proteins, the automation of protein function prediction (PFP) using computational tools is critical ^1, 2^. However, diverse data sources, data quality issues, like noise and incompleteness, and a lack of consensus on the best predictor(s) for various types of data and functions pose serious challenges for PFP. Specifically, data types used by existing PFP methods have included amino acid sequences, protein structure information, gene expression profiles and protein-protein interaction networks. Similarly, prediction methodologies have ranged from homology-based sequence alignment to machine learning algorithms, network-based methods, and others. Several community-based critical assessments, especially CAFA ^3, 4^, have been organized to objectively measure the performance of these diverse PFP methods. A central finding from these assessments was the variable performance of the tested methods/predictors for different functional terms from the Gene Ontology (GO) ^5, 6^ and target proteins, demonstrating that there is no ideal predictor of all types of protein function.

A potential approach for improving prediction performance in such a scenario of diverse data types and individual/base predictors is to build *heterogeneous ensembles* ^7^. These ensembles harness the consensus and diversity among the base predictors, and can help reduce potential overfitting and inaccuracies incurred by them. Unsupervised methods like majority vote and mean aggregation, and supervised approaches like stacking and ensemble selection are the most commonly used methods for building heterogeneous ensembles. Stacking builds such an ensemble by learning a function, also known as a meta-predictor, that optimally aggregates the outputs of the base predictors ^8^. Ensemble selection methods iteratively add one or more base predictors to the current ensemble either greedily or to improve the overall diversity and performance of the ensemble ^9– 11^. These approaches have been successfully applied to a variety of prediction problems ^12– 15^.

In previous work ^7^, we tested the efficacy of heterogeneous ensembles for annotating approximately 4,000 *Saccharomyces cerevisiae* proteins with GO terms. For this, we evaluated stacking using logistic regression as the meta-predictor and Caruana *et al.*’s ensemble selection (CES) algorithm ^9, 10^, both implemented in our open-source package DataSink. The implementation uses a nested cross-validation setup ^7^ to train the base predictors and the ensembles independently with the aim of reducing overfitting ^16^ and improving prediction performance. These experiments yielded that both CES and stacking performed significantly better than stochastic gradient boosting ^17^, the best-performing base predictor for all the GO terms considered. This improvement was observed both in terms of the AUC score, as well as the *F*_max_ measure, which has been established to be more relevant for PFP evaluation ^3, 4^.

A major limitation of this previous study was the relatively high computational cost of constructing heterogeneous ensembles, despite their high-performance computing (HPC)-enabled implementations in DataSink. Due to this cost, we were able to test the ensembles’ performance on only three GO terms for proteins of only one organism ( *S. cerevisiae*). Owing to the same limitation, only logistic regression was tested as the meta-predictor for stacking. Thus, despite the initial encouraging results, it remains unclear if heterogeneous ensembles provide the same improvement over individual base predictors for a substantial part of GO as well as for a large number of proteins from multiple organisms.

The overall goal of this study is to critically assess this ability of heterogeneous ensembles to improve PFP at a large scale across a multitude of functional terms, proteins and organisms. For this, we adopt an HPC-enabled strategy to evaluate heterogeneous ensembles, built using CES and stacking with eight meta-prediction algorithms, for large-scale PFP. This evaluation is conducted over 277 GO terms, and more than 60,000 proteins, from 19 pathogenic bacterial species. Specifically, we analyze the following aspects of of heterogeneous ensembles:

1. Prediction performance compared to that of the best-performing individual predictor for each GO term.
2. How this performance varies for different GO terms categorized by:
  (a) Number of genes annotated to each term (size).
  (b) Different depths in the GO hierarchy (levels of specificity).

We expect the results of this study to shed light on the efficacy of heterogeneous ensembles for large-scale protein function prediction. To enable the application of these ensembles to other related problems, we have publicly shared the HPC-enabled code underlying this work as LargeGOPred.

## Methods

### Data used in the study

We extracted the amino acid sequences of 63,449 proteins from 19 clinically relevant bacterial pathogens, which include a subset of organisms from the Health and Human Services (HHS) list of select agents and those with current high clinical relevance ^18, 19^. The annotations of these proteins to GO terms used in this study were either inferred by a curator (evidence codes: ISS, ISO, ISA, ISM, IGC, IBA, IBD, IKR, IRD, RCA, TAS, NAS and IC) or from experiments (evidence codes: EXP, IDA, IPI, IMP, IGI and IEP), but not from electronic annotations (IEA) in the UniProt database ^20^. We selected 277 molecular function (MF) and biological process (BP) GO terms with more than 200 annotated proteins across all the 19 bacteria. The constantly changing contents of the GO ontology and annotations, as well as our incomplete knowledge of the latter make it possible for sequences not annotated to a GO term to be annotated in the future. Thus, to prepare more well-defined datasets, for each GO term, we defined proteins annotated to it as positive samples and any proteins that are neither annotated to the GO term nor its ancestors or descendants as negative samples ^21^. The resultant distributions of GO terms with regard to the number of proteins positively annotated to them for each organism and across all organisms are shown in Table 1.

**Table 1.** Overview of the data used in this study. The ‘#Proteins’ column shows the number of proteins in the corresponding bacterial pathogen listed in the ‘Organism’ column. The disease(s) each of these pathogens has been implicated in are listed in the ‘Disease(s)’ column. The ‘Distribution of GO terms’ column with 3 sub-columns shows the number of proteins annotated with GO terms with that range of #annotations, with the corresponding number of GO terms shown in parenthesis. The final row of the table shows the total number of proteins and GO terms considered in this study. Ranges of distributions of GO terms for all species are shown in the parenthesis of the three ‘#annotations’ sub-columns. Since each GO term is considered independently, each protein may be counted as annotated to multiple GO terms.

Organism	Disease(s)	#Proteins	Distribution of GO terms (#annotations)		
			0-10 (200-500)	10-100 (500-1000)	>100 (>1000)
*Yersinia pestis*	plague, black death	7375	164 (26)	7397 (218)	6773 (33)
*Mycobacterium tuberculosis*	tuberculosis (TB)	6112	53 (12)	8850 (186)	19095 (79)
*Burkholderia vietnamiensis*	severe respiratory disease	4889	49 (277)	0	0
*Pseudomonas aeruginosa*	nosocomial infection	4488	44 (6)	8515 (171)	23891 (100)
*Klebsiella pneumoniae*	nosocomial infection, pneumonia	4140	66 (277)	0	0
*Escherichia coli*	severe abdominal cramps	4067	1 (1)	6811 (104)	53731 (172)
*Vibrio cholerae*	cholera	3756	100 (13)	8218 (164)	27961 (100)
*Salmonella typhimurium*	gastroenteritis	3713	64 (11)	8861 (224)	9532 (42)
*Shigella dysenteriae*	shigellosis	3039	69 (277)	0	0
*Peptoclostridium difficile*	pseudomembranous colitis	2925	168 (277)	0	0
*Bordetella pertussis*	pertussis or whooping cough	2688	123 (277)	0	0
*Clostridium botulinum*	botulism poisoning	2678	277 (64)	5609 (191)	4076 (22)
*Enterococcus faecium*	neonatal meningitis or endocarditis	2343	0 (277)	0	0
*Staphylococcus aureus*	severe skin infections	2142	415 (72)	5628 (184)	3863 (21)
*Acinetobacter baumannii*	nosocomial infection	1946	0 (277)	0	0
*Haemophilus influenzae*	bacteremia, pneumonia	1500	526 (79)	5233 (178)	3947 (20)
*Neisseria gonorrhoeae*	sexually transmitted disease	1464	141 (270)	175 (7)	0
*Streptococcus pyogenes*	pharyngitis, impetigo	1332	154 (277)	0	0
*Helicobacter pylori*	peptic ulcers, gastritis, stomach cancer	1145	374 (272)	217 (5)	0
***Total***		**63449**	**47226 (152)**	**51720 (71)**	**122225 (54)**

We chose normalized k-mer frequencies, extracted using the khmer package (2.1.1) ^22^, as our feature set to represent the information contained in the amino acid sequences and construct a feature matrix that can serve as input for LargeGOPred. K-mers have been used for similar purposes in several PFP studies ^1^, as well as related problems like the prediction of protein secondary structure ^23^ and RNA-protein interactions ^24^. Since the size of the feature set (all possible k-mers) grows rapidly with increasing value of k, setting k to a high value may be impractical for large-scale PFP tasks like ours. Additionally, 1- and 2-mers may not provide enough context information about the sequence. Thus, we set k = 3 since this value strikes a balance between the information captured by the k-mers and computational scalability. For each amino acid sequence, we extracted frequencies for all possible 8,000 3-mers at each position of the sequence. We then normalized these frequencies by the length of the sequence to reduce the potential bias due to the variation of sequence lengths among the proteins.

All the processed data are available from https://zenodo.org/record/1434450#.W6lU2hNKhBx (doi: 10.5281/zenodo.1434450) ^25^.

### Overview of the prediction approach

**Figure 1.**
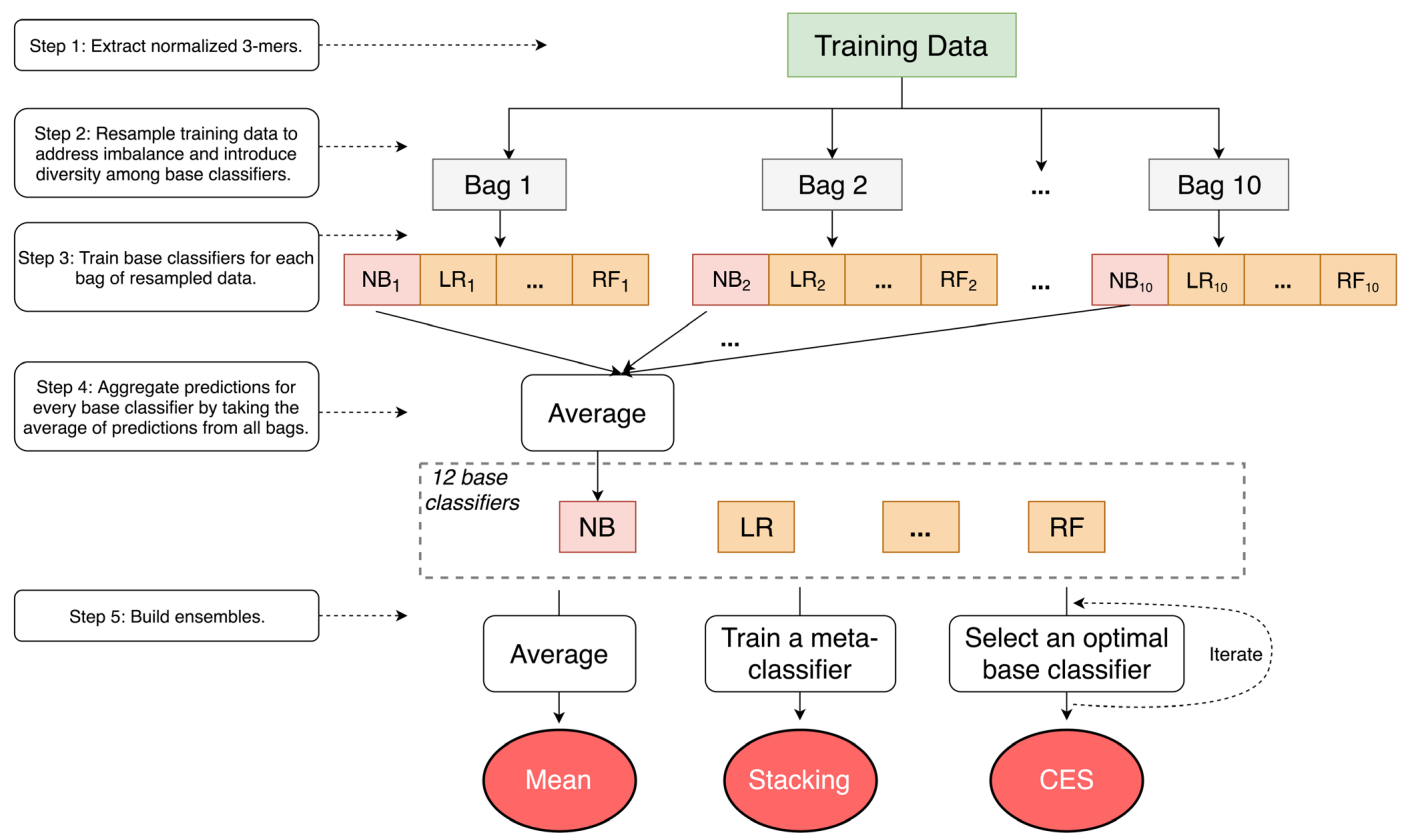
Overview of the prediction approach. We first extracted normalized 3-mer frequencies from the amino acid sequences as features. Training data for 12 types of base classifiers (upper half of Table 2) were randomly under-sampled into 10 bags containing equal numbers of positive and negative samples to address class imbalance and to introduce diversity among base classifiers, even among those of the same type. The predictions from these bags were averaged for each base classifier and collected to train the heterogeneous ensembles using three types of methods, namely mean aggregation, 8 stacking meta-classifiers (bottom half of Table 2), and Caruana *et al.*’s ensemble selection (CES). Separate test data were used to evaluate the heterogeneous ensembles. The entire process was conducted within a nested cross-validation setup (described below) executed for each target GO term separately.

The overall approach adopted for this study is visualized and described in detail in Figure 1. Two key components of the approach, specifically the heterogeneous ensemble methods used and nested cross-validation, are described in the following subsections, as well in our previous work ^7^. The prediction performance of all the predictors tested in this study, specifically the base classifiers and ensembles, was evaluated in terms of the *F*_max_ measure, which is the maximum value of F-measure ^26^ across all binarization thresholds, and has been recommended as a PFP evaluation measure by CAFA ^3, 4^. We also evaluated the statistical significance of the difference between the performance of the various predictors (described below) ^27^. Finally, since we approach GO term prediction as a binary classification problem, the terms “predictor” and “classifier”, and their variants will be used interchangeably as appropriate in the rest of the paper.

**Table 2.** Base classifiers used to construct all the heterogeneous ensemble methods tested in this study (upper half), and meta-classifiers used to construct stacking-based ensembles (lower half). The base and meta-classifiers were adopted from Weka ^28^ and scikit-learn ^30^ respectively.

Base classifiers	
Classifier name	Weka class name
Naive Bayes (NB)	*weka.predictors.bayes.NaiveBayes*
Logistic Regression (LR)	*weka.predictors.functions.Logistic*
Stochastic Gradient Descent (SGD)	*weka.predictors.functions.SGD*
Voted Perceptron (VP)	*weka.predictors.functions.VotedPerceptron*
AdaBoost (AB)	*weka.predictors.meta.AdaBoostM1*
Decision Tree (DT)	*weka.predictors.trees.J48*
Logit Boost (LB)	*weka.predictors.meta.LogitBoost*
Random Tree (RT)	*weka.predictors.trees.RandomTree*
Random Forest (RF)	*weka.predictors.trees.RandomForest*
RIPPER	*weka.predictors.rules.JRip*
PART	*weka.predictors.rules.PART*
K-nearest Neighbors (KNN)	*weka.predictors.lazy.IBk*
Meta-classifiers	
Meta-classifier	Scikit-learn class name
Naive Bayes (NB)	*sklearn.naive_bayes.GaussianNB*
AdaBoost (AB)	*sklearn.ensemble.AdaBoostpredictor*
Decision Tree (DT)	*sklearn.tree.DecisionTreepredictor*
LogitBoost (LB)	*sklearn.ensemble.GradientBoostingpredictor*
K-nearest Neighbors (KNN)	*sklearn.neighbors.KNeighborspredictor*
Logistic Regression (LR)	sklearn.linear_model.LogisticRegression
Stochastic Gradient Descent (SGD)	sklearn.linear_model.SGDpredictor
Random Forest (RF)	*sklearn.ensemble.RandomForestpredictor*

### Heterogeneous ensemble methods

We used 12 diverse base predictors from the Weka machine learning suite (3.7.10) ^28^ (upper half of Table 2) and built 3 types of unsupervised and supervised heterogeneous ensembles on top of them. The unsupervised mean method simply takes the average of the predictions from base classifiers as the final prediction. For supervised heterogeneous ensembles, we tested various stacking methods and one of the most widely used ensemble selection methods, namely CES.

***Stacking.*** Stacking builds a heterogeneous ensemble by learning a meta-classifier that optimally aggregates the outputs of the base predictors. Unlike our previous study, where only stacking using logistic regression as the meta-classifier was tested, we used 8 different meta-classifiers in this study (bottom half of Table 2), and statistically compared their performance over all the target prediction problems.

***Ensemble selection and CES.*** Ensemble selection is a process to selecting a subset of all the base classifiers that are mutually complementary such that the resultant ensemble is as predictive as possible.

In this study, we tested Caruana *et al*’s ensemble selection (CES) algorithm for large-scale PFP ^9, 10^. CES is an iterative algorithm that starts with an empty ensemble, and in each iteration, adds the base predictor that best improves the resultant ensemble’s performance, partly due to the added predictor’s complementarity to the current ensemble. The process continues until the ensemble’s performance doesn’t improve anymore, or even starts decreasing. In this work, we tested the version of CES in which the base predictor to be added to the ensemble was sampled with replacement in each iteration ^9^.

### Nested cross-validation

Cross validation (CV) is a frequently used methodology for training and testing classifiers and other predictors ^29^. However, in the case of learning supervised ensembles like ours that involve two rounds of training (first the base classifiers and then the ensembles), using standard cross-validation may lead to overfitting of the ensemble. Thus, as explained in our previous work ^7^, we devised a nested cross-validation procedure to be used for training and testing supervised ensembles. In this procedure, the entire dataset was split into *outer* training and test CV splits and each outer training split was further divided into *inner* CV folds. Base classifiers were trained on the inner training split and used to predict on the corresponding inner test split. Predictions made by the base classifiers were collected across all inner testing folds and used as the base data to train the heterogeneous ensembles. The outer test splits were then used to evaluate the performance of the trained ensembles. The nested cross-validation strategy ensures that the base classifiers and ensembles are trained on separate subsets of the data set, thus reducing the chances of bias and overfitting.

We addressed the potentially high computational costs by parallelizing all the independent units of the nested CV process, namely the training and testing of base and ensemble predictors over all the inner and outer CV splits. These units were then executed on separate processors in a large HPC cluster, with the outputs of inner CV folds flowing into the outer ones as described in our earlier work ^7^. We have made this HPC-enabled implementation of the heterogeneous ensemble PFP process publicly available as LargeGOPred.

### Statistical comparison of PFP performance

In this study, we compared multiple heterogeneous ensembles and base classifiers on their ability to predict annotations to a large number of GO terms. In such situations, it is critical to assess the statistical significance of these numerous comparisons to derive reliable conclusions. For this, we used Friedman’s and Nemenyi’s tests and visualized their results in easily interpretable critical difference (CD) diagrams ^27^. Friedman’s test ranks all the tested classifiers over all datasets (here, GO terms) and tests if the mean ranks of all classifiers are statistically equivalent, while Nemenyi’s test performs the equivalent of multiple hypothesis correction for these comparisons. We used the scmamp (0.3.2) ^31^ R package to perform these tests and visualize their results as CD diagrams.

## Results

### Overall PFP performance

**Figure 2.**
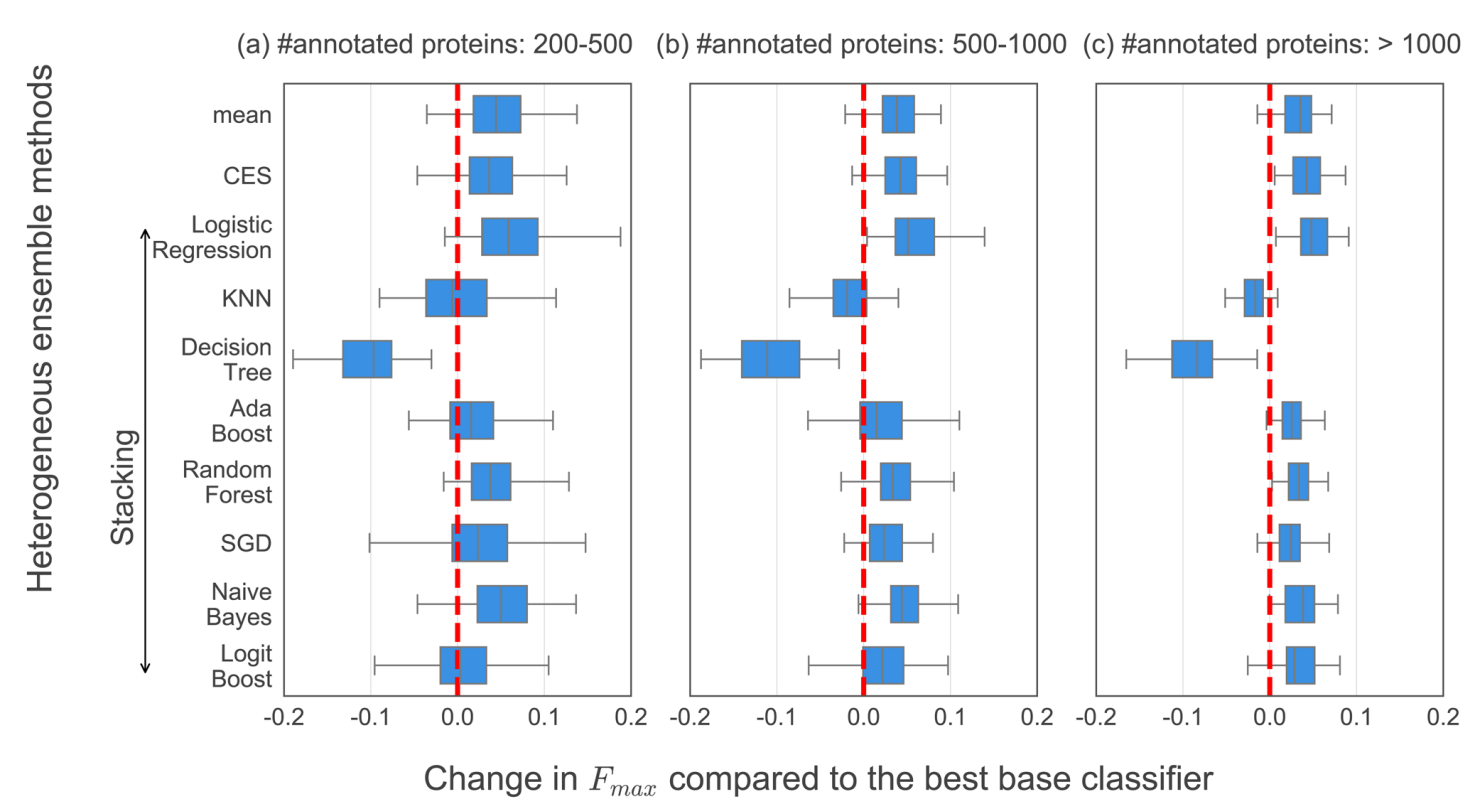
Boxplots denoting the distributions of the heterogeneous ensembles’ PFP performance compared to that of the best base classifier for each GO term. The Y-axis shows all heterogeneous ensembles tested, specifically mean (aggregation), Caruana *et al.*’s ensemble selection (CES) and 8 stacking methods using different meta-classifiers named here. The X-axis denotes the difference between the *F*_max_ of each heterogeneous ensemble and the best base classifier for each GO term (∆ *F*_max_), which are categorized into ( **a**) 152 small, ( **b**) 71 medium and ( **c**) 54 large GO terms with 200-500, 500-1000 and over 1000 annotated sequences in our dataset ( Table 1). The broken vertical red line in each subplot represents ∆ *F*_max_=0.

We first evaluated if and to what extent heterogeneous ensembles enable the prediction of protein function as compared to individual predictors. Figure 2 shows the results of this evaluation in terms of the difference of the performance of a variety of ensembles from that of the best base classifier for each GO term, with the terms themselves categorized by their sizes. Although there is substantial variability in the values of ∆ *F*_max_ across ensemble methods and GO term categories, some trends can still be observed. First, the values of ∆ *F*_max_ across ensembles increase as the sizes of the GO terms considered also increase. This is illustrated by the fact that zero, one (Stacking with Logistic Regression) and four (CES and Stacking with Logistic Regression, Random Forest and Naive Bayes) ensembles produce ∆ *F*_max_>0 for every GO term tested in the small, medium and large categories (from left (a) to right (c) in Figure 2). This trend is expected, since the availability of more positively annotated genes in the larger GO terms enhances the ability of the ensembles, especially the supervised ones, to improve PFP performance. Due to the same reason of more training data, the variability of PFP performance for the large terms, represented by the widths of the boxes and whiskers, is smaller, illustrating increased robustness of the ensembles.

**Figure 3.**
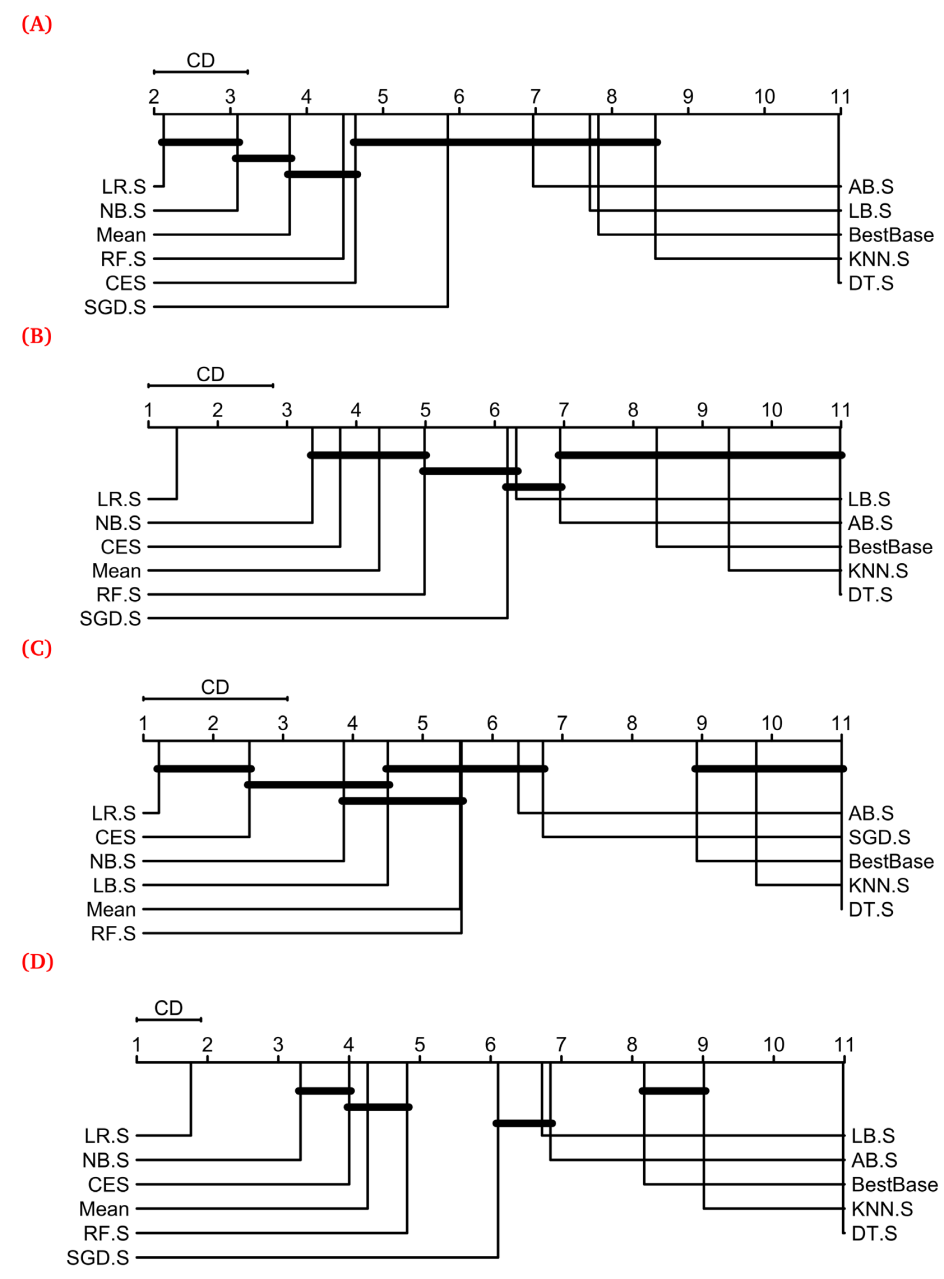
Critical Difference (CD) diagrams showing the results of a statistical comparison of the performance of all the heterogeneous ensemble methods shown in Figure 2 and the best base classifier for each GO term, conducted using Friedman and Nemenyi’s tests ^27^. In these diagrams, PFP methods, represented by vertical+horizontal lines, are displayed from left to right in terms of the average rank obtained by their resultant models for each GO term included. The groups of methods producing statistically equivalent performance are connected by horizontal lines. ( **A**)–( **C**) show the CD diagrams for the three categories of GO terms shown in Figure 2, while ( **D**) shows the one for all the 277 GO terms considered in this study. The *scmamp* R package ^31^ was used to perform the Friedman and Nemenyi’s tests and plot the CD diagrams. Meta-classifiers used within stacking are denoted by their commonly used acronyms, e.g. LR for Logistic Regression, appended with “.S”.

To analyze these results in further detail and derive reliable conclusions from them, we used Friedman’s and Nemenyi’s tests to statistically assess the ∆ *F*_max_ values shown in Figure 2. Figure 3 shows the results of these tests visualized as Critical Difference (CD) diagrams for the three categories of GO terms shown in Figure 2A–C, as well as all of them taken together ( Figure 2D). These results show that several heterogeneous ensemble methods, such as LR.S, NB.S, Mean, RF.S, CES and SGD.S, performed better than the respective best base classifier in terms of their average rank ^27^. In contrast, KNN.S and DT.S performed worse than the best base classifier for each category of GO terms considered.

A consistent observation from Figure 3 is that Stacking using Logistic Regression (LR.S) performed the best among all the tested predictors (leftmost entry in the CD diagrams) regardless of the GO term category considered. It performed statistically equivalently with NB.S and CES for the small ( Figure 3A) and large ( Figure 3C) GO terms respectively, statistically confirming the observations made from Figure 2. In particular, LR.S exclusively performed the best among all the predictors over all the GO terms examined, consistent with its good performance over a limited number of GO terms in our previous work ^7^. Thus, we further analyzed the performance of this predictor across the hierarchical structure of the Gene Ontology.

### Performance of Stacking using Logistic Regression (LR.S) across the GO hierarchy

**Figure 4.**
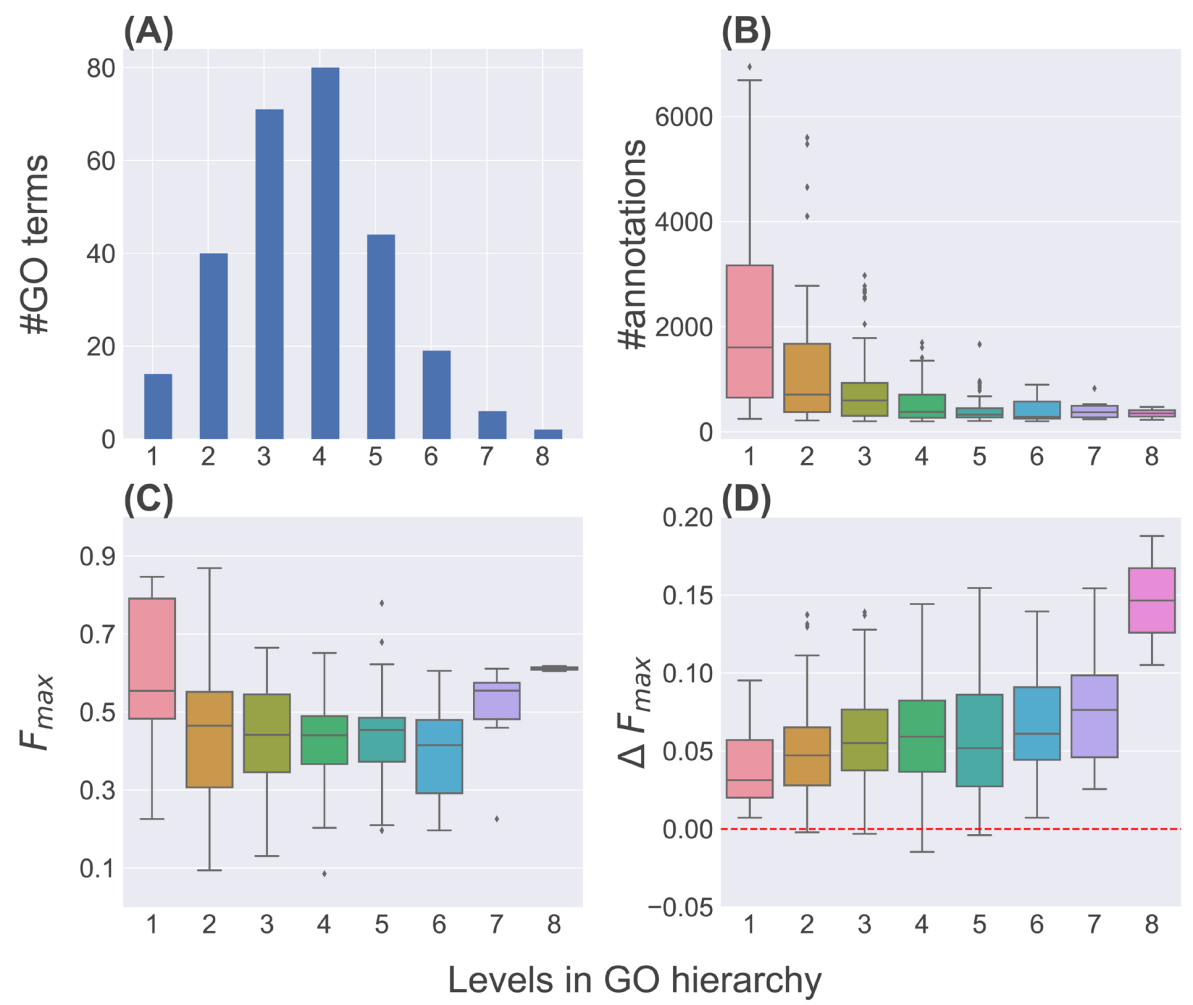
Performance of Logistic Regression (LR.S) for terms at different levels of the GO hierarchy. ( **A**) and ( **B**) show the distributions of the number of GO terms and the number of genes annotated to these terms at different levels respectively. ( **C**) and ( **D**) show the distributions of LR.S’s *F*_max_ scores and their differences from the corresponding scores of the best classifier (∆ *F*_max_) for these GO terms at the various levels.

GO terms are not a flat set of labels, but are rather organized in hierarchical ontologies structured as directed acyclic graphs (DAGs) ^5, 6^. Terms vary in their depth, or level, with deeper terms representing more specific functions as compared to those at shallower levels. Using the definition of the level of a GO term as the length of the shortest path to it from the root of the hierarchy, implemented in the GOATOOLS python package (0.8.4) ^32^, we observed that the levels of the terms in our dataset varied between 1 and 8 ( Figure 4(A)). In terms of the number of genes annotated, as expected, most of the annotations are to the shallower GO terms and only a small number to the deeper ones ( Figure 4(B)).

We analyzed the ability of LR.S to predict annotations to these terms, measured in terms of *F*_max_, at different levels ( Figure 4(C)). The performance is reasonably high at level 1, but decreases gradually until level 6 due to fewer annotations available for training the base classifiers and ensembles ( Figure 4(B)). The performance improves slightly at levels 7 and 8, likely due to the increased specificity of the corresponding terms and thus better signal in the corresponding training data.

Finally, we analyzed how LR.S’s performance compared with that of the best classifier for the tested GO terms at different levels of the hierarchy. For this, we calculated and plotted in Figure 4(D) the same ∆ *F*_max_ measure shown in Figure 2, this time categorized by levels. The results in Figure 4(D) show that ∆ *F*_max_ increases overall for GO terms at increasingly deeper levels in the hierarchy. The increases are statistically significant (Wilcoxon rank-sign test p-value<0.05) at levels 1–7, although not significant (p-value=0.17) for only two terms at level 8 ( Figure 4(A)). These results indicate the benefit heterogeneous ensembles, specifically LR.S, can provide for deeper GO terms with fewer annotations where individual predictors may not be effective.

## Discussion

Owing to the diversity of available data types and computational methodologies, a variety of methods have been proposed for protein function prediction (PFP) ^1, 2^. CAFA ^3, 4^ and other large-scale assessment efforts demonstrated that there is no ideal method for predicting different types of functions. In this paper, we have demonstrated a potential approach to address this problem, namely assimilating individual methods/predictors into heterogeneous ensembles that may be more robust, generalizable and predictive across functions. Although we had provided preliminary results supporting this approach in our previous work ^7^, those results were limited to predicting annotations to only three GO terms. In this paper, we report the first comprehensive and large-scale assessment of protein function prediction using heterogeneous ensembles. Specifically, using a data set of over 60,000 bacterial proteins annotated to almost 300 GO terms, we assessed how the mean aggregation, CES and stacking using multiple meta-classifiers performed for PFP.

Several of the tested heterogeneous ensembles performed better than the best base/individual predictor for many of the GO terms examined. In particular, the performance improvements obtained by heterogeneous ensembles generally increased with more annotations available for a given GO term, i.e. its size, which can be expected due to the larger amount of more positive data available for training the base predictors and ensembles.

A rigorous statistical comparison of all the heterogeneous ensembles and best base predictors tested over different categories of GO terms based on their sizes reaffirmed the effective performance of ensembles for PFP. In particular, Stacking using Logistic Regression (LR.S) was consistently the best-performing ensemble method across all the GO term categories, a finding consistent with our earlier work ^7^. The effectiveness of LR.S can be attributed to the simplicity of the logistic regression function, which can help control overfitting at the meta-learning level during stacking. This effectiveness was also reflected in our observation that LR.S’s is increasingly more accurately predictive for GO terms deeper in the hierarchy, for which the small number of annotations available may adversely affect individual predictors. Overall, our study and results demonstrate the potential of heterogeneous ensembles to advance protein function prediction on top of the progress in individual predictors already being reported in CAFA ^3, 4^ and other exercises.

A key feature of our work was the effective utilization of high-performance computing (HPC) to enable efficient large-scale PFP. Specifically, using a large number processors in a sizeable HPC cluster, we successfully built and evaluated heterogeneous ensembles for over 60,000 bacterial proteins annotated to almost 300 GO terms in under 48 hours. While this increase in efficiency is already appreciable, it can be improved further by utilizing more parallelized formulations of the process, such as using parallel implementations of base classification methods ^33^ instead of the serial versions used in this work.

Although the results of our study are encouraging, they were derived using data from only 19 pathogenic species due to our group’s general interest in PFP to better understand and predict annotated and unannotated pathogenicity in the context of clinically relevant bacteria. The inclusion of a larger number of and more diverse species, both prokaryotic and eukaryotic, in this evaluation can help assess how well our methods generalize to other species. The same can be said for including other types of data as well, such as the gene expression profiles used in our previous work ^7^.

We also only used normalized k-mer frequencies derived from amino acid sequences to represent proteins. This could be extended to test other representations such as short linear motifs (SLiMs) ^34^, hidden Markov models (HMMs) ^35^ and learned protein embeddings ^36^. Moreover, regardless of the representation, another potential issue is that highly conserved and thus similar sequences across the 19 species tested in this study might be separated into both the training and test sets, which may result in an overestimation of prediction performance. Though UniProt controls for within species redundancy, it does not remove redundancy between species, an issue also true for our dataset. To address this issue, non-redundant versions of UniProt, such as UniRef100 or UniRef90 ^20^, could be used to design more representative training and test sets. However, since the same prediction and evaluation process is used throughout our study, this issue should not adversely affect the fairness of the comparison between the performance of base predictors and heterogeneous ensembles.

Finally, in this study, we considered GO terms as independent units of protein function, but they are actually related because of their organization in the hierarchical structure of GO. Information from ancestors and closely related siblings in the hierarchy may provide useful information for protein function prediction, including through heterogeneous ensembles. Previous work has utilized this information for advancing individual and ensemble PFP algorithms ^37– 39^, and similar ideas can be used to improve heterogeneous ensembles as well.

## Data availability

The data underlying this study is available from Zenodo. Dataset 1: Data for LargeGOPred. http://doi.org/10.5281/zenodo.1434450 ^25^

This dataset is available under a Creative Commons Attribution 4.0

## Software availability

Source code underlying this work is available from GitHub: https://github.com/GauravPandeyLab/LargeGOPred

Archived source code at time of publication http://doi.org/10.5281/zenodo.1434321 ^40^

License: GNU General Public License, version 2 (GPL-2.0)).
