# Supplementary material for: Large-scale protein function prediction using heterogeneous ensembles
Source: F1000Res. 2018 Sep 28;7:ISCB Comm J-1577. [Version 1] doi: 10.12688/f1000research.16415.1 (PMC6221071; doi:10.12688/f1000research.16415.1)
Supplement: Peer review history

# Peer review: Referee response for version 1

**DOI:** 10.5256/f1000research.17934.r38881

**Published:** 2018-11-05

**Authors:** Predrag Radivojac

**Affiliations:** 1 College of Computer and Information Science, Northeastern University, Boston, MA, 02115, USA

This study evaluates protein function prediction using heterogeneous ensembles. The authors collected a set of 19 organisms with functional annotations and used a complex cross-validation setup to explore the value of obtaining improved classification performance using model averaging, stacking, and previously proposed techniques by Caruana et al. They considered 277 binary classification problems, each with its own data set of positive and putatively negative genes. The base classifiers were built upon a simple 3-mer feature representation.

 Overall, this work is well presented and is clear in its exposition and contributions: there is value in developing heterogeneous ensembles though the computational cost is significant (here, an HPC solution was necessary to complete the study). Simple stacking models with logistic regression seem to be performing the best. This comes as a small surprise because one would expect nonlinear models to have an edge. On the other hand the base models were already nonlinear which might contribute to this effect.

 Software for this work is available which is a plus.

 Specific comments:

1. (the basis for answering one of the questions with "partly") Page 3, "Data used in the study"

The authors say that no electronic annotations have been used, but the majority of the evidence codes provided is in fact electronic annotation. See

 http://www.geneontology.org/page/guide-go-evidence-codes

 Some of the results of this work might be less realistic if the models were trained on predicted annotations. On the other hand, given the state of annotation of bacterial genomes, it is not clear whether there was an alternative. Nonetheless, this requires clarification, discussion and changes in this paragraph or perhaps elsewhere too. 

       2. The authors refer to their previous work on the inner and outer cross-validation folds. Although I believe I understood the process, it would be useful to mention whether at any point a base classifier was trained on a particular protein and then the stacked model included that same protein in its training. 

        3. Figure 1, lower part, ended up not being useful for me. Once we train an ensemble of base classifiers in step 3, I was confused by step 4. This seems to be some intermediate averaging that comes before stacking. This point would be good to explicitly point to the reader as it confused me at one point.

        4. Not a mandatory request, but it would be useful to perform a leave-one-species-out type of accuracy estimation. This might combat the problems related to sequence similarity that are discussed near the end of the paper. It would also provide evidence on what to expect from computational models when a new species is sequenced.

         5. The manuscript would greatly benefit from proofreading and learning up some sentence structure and language issues.

I have read this submission. I believe that I have an appropriate level of expertise to confirm that it is of an acceptable scientific standard.

# Peer review: Referee response for version 1

**DOI:** 10.5256/f1000research.17934.r38879

**Published:** 2018-10-24

**Authors:** Guoxian Yu

**Affiliations:** 1 College of Computer and Information Sciences, Southwest University, Chongqing, China

This paper investigates the potential of heterogeneous ensembles for protein function prediction by quantitatively comparing several classical base classifiers and ensembles on them. This investigative study is interesting, innovative and informative for future study on protein function prediction. This manuscript is clearly presented, well designed and organized. This investigation can be further improved in the following aspects:

1. The used data are only Amino Acid sequences, will the results and conclusions be changed when other types of data are used and integrated? The heterogeneous ensembles are intended for heterogeneous data types.
2. The considered GO terms (annotated to 200-300 proteins) are quite small, compared with the large GO terms space, more specific GO terms (annotated to <200 and >=10 proteins) should be tested. PFP is an imbalanced function prediction problem.
3. Smin is another more stringent evaluation metric in CAFA, and it refers to GO hierarchy when measuring the performance. This metric should be additionally used to quantify the performance of PFP.
4. There are some classifier ensemble based PFP solutions omitted. They should be cited and acknowledged.

I have read this submission. I believe that I have an appropriate level of expertise to confirm that it is of an acceptable scientific standard.
